# Control Shear Banding in Metallic Glasses to Enable Tensile Ductility: A Brief Review

**DOI:** 10.3390/ma19122679

**Published:** 2026-06-22

**Authors:** Shan Li, Saisai Zhang, Xiushuo Zhang, Jingli Sun, Haiyang Song

**Affiliations:** 1 National Key Laboratory of Solid Rocket Propulsion, Rocket Force University of Engineering, Xi’an 710025, China; 2 School of New Energy, Xi’an Shiyou University, Xi’an 710065, China

**Keywords:** metallic glasses, shear band, tensile ductility, structural heterogeneity, toughening strategies

## Abstract

Metallic glasses (MGs) exhibit excellent mechanical properties, yet their poor tensile ductility greatly limits their practical applications as structural and functional materials. Shear banding is a typical localized rheological deformation behavior inherent to amorphous materials, which stems from heterogeneous atomic rearrangement and regional viscosity fluctuations in the glassy matrix, and fundamentally determines the macroscopic mechanical properties of MGs and their composites. This review discusses the relationship between typical toughening strategies and shear banding behavior, and proposes that deliberate suppression of shear band (SB) initiation or deceleration of their rapid propagation can effectively promote distributed plastic flow. In this review, nanosizing and metamaterial strategies are shown to hinder the formation of mature SBs, while metallic glass matrix composites (MGMCs), nanoglasses (NGs), notched design, and rejuvenation treatments contribute to restraining SB propagation. Current approaches have successfully regulated shear banding behavior and thereby realized appreciable tensile ductility in MGs. Novel design and fabrication techniques for amorphous alloys, which suppress SB initiation and retard SB propagation to achieve homogeneous plastic flow, open up new avenues for realizing controllable plasticity of MGs.

## 1. Introduction

Metallic glasses (MGs), first discovered nearly 60 years ago, are solid metals or alloys that retain a highly disordered atomic structure inherited from the liquid state [1]. Amorphous alloys must be rapidly quenched from supercooled liquids to avoid crystallization, a processing constraint that originally limited MG products to low-dimensional forms (geometric structures with one or two dimensions far smaller than the third), such as ribbons, foils, or wires [2]. With rapid advances in materials processing techniques, Chen employed the suction-casting method to fabricate MGs with a size of 1 mm at moderate cooling rates [3]. Since then, glassy alloys with millimeter-scale thickness have been defined as bulk metallic glasses (BMGs) [4,5,6]. To date, the largest BMG possesses a diameter of 80 mm and a length of 85 mm [7]. BMGs exhibit ultrahigh strength but lack strain-hardening capacity and typically demonstrate strain-softening behavior [8,9,10]. Consequently, conventional BMGs display negligible global tensile ductility at room temperature, severely restricting their widespread applications in structural and functional engineering fields [11,12,13,14,15,16]. Tensile ductility refers to the intrinsic mechanical property that enables MG materials to undergo continuous and uniform plastic deformation under uniaxial tensile loading prior to final fracture, which is a crucial criterion for assessing their structural service reliability.

Owing to the absence of mobile dislocations in amorphous atomic configurations, the fundamental unit of plastic deformation in MGs is shear transformation (ST) [17,18,19,20]. However, such a microscopic plastic deformation mechanism cannot guarantee macroscopic ductility under tension [21,22,23]. The underlying reason is that the accumulation and percolation of ST events generate shear bands (SBs), which propagate rapidly without resistance from crystalline defects and eventually lead to catastrophic brittle failure [8,17,18]. By definition, ST refers to the cooperative rearrangement of atomic groups to overcome energy barriers, and the regions accommodating these rearrangements are termed shear transformation zones (STZs) [15]. The lack of tensile ductility remains a major bottleneck limiting the practical engineering application of high-strength BMGs. To address this issue, numerous advanced toughening strategies have been proposed in recent decades [15,24,25]. Toughening strategies represent a category of systematic structural design and optimization methodologies tailored for MGs, which function to regulate shear banding behaviors, alleviate localized strain concentration, and effectively improve the tensile ductility and overall fracture resistance of MG materials. Ma et al. proposed the “four R’s” strategy including Relaxation, Refinement, Retention and Rejuvenation in a review [15]. This framework focuses on modulating atomic configurations, structural inhomogeneities and energy states of MGs, and summarizes the intrinsic ductility potential achievable by tailoring the amorphous structure. Shear localization originates from the intrinsic structural heterogeneity of amorphous alloys, leading to severely unstable plastic deformation in the form of shear banding. Under applied stress, shear deformation induces excessive free volume and increased structural disorder, triggering spontaneous shear localization. These localized regions are rich in fragmented clusters (geometrically unfavored motifs), which are energetically favorable for ST activation. As a result, conventional MGs show almost no measurable plastic strain under tension. Therefore, deliberately tuning the internal structural heterogeneity of amorphous alloys to expand the achievable ductility range is of great scientific and engineering significance. The purpose of this review is to highlight several typical toughening strategies that have broadened the ductility envelope of these high-strength materials.

Our core perspective is that deliberate control of shear banding—especially suppressing SB formation—can rejuvenate the glassy structure into a more deformable state. This review is compiled based on a systematic literature retrieval and screening process. Relevant publications were mainly collected from mainstream academic databases including Web of Science and ScienceDirect. The primary search keywords adopted are MG, SB, tensile ductility, toughening strategy and structural rejuvenation. During literature selection, we retained peer-reviewed original research articles and classic review papers focusing on the deformation mechanism and performance regulation of MG s, while excluding documents with irrelevant themes, repeated content or insufficient research credibility. After retrieval, preliminary screening, in-depth reading and content classification, we conducted a comprehensive summary and discussion of existing research findings.

The overall structure of this review is organized as follows: Section 2 elaborates the correlation between structural heterogeneity and shear banding evolution; Section 3 systematically summarizes typical advanced toughening strategies for BMGs; and Section 4 concludes the current progress and proposes prospective research directions for the future development and application of amorphous alloys.

## 2. The Plastic Deformation of MGs

### 2.1. Structural Evolution

**Figure 1:**
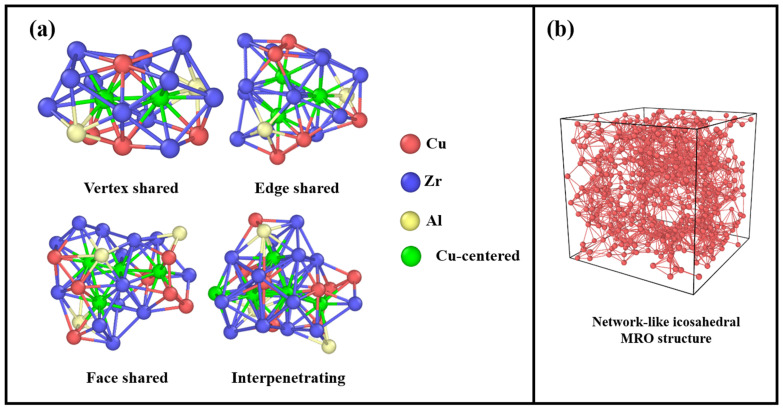
(**a**) The icosahedral atomic configuration and the characteristic atomic clusters constructed by interpenetrating icosahedra; (**b**) Sketch of microstructure of BMGs.

The exceptional mechanical performance and intrinsic room-temperature brittleness of MGs originate fundamentally from their unique disordered atomic structures [26,27,28,29]. Diverse local atomic packing environments arise from the structural heterogeneity of amorphous alloys, leading to variable mechanical properties [15,30,31,32,33]. Atoms in MGs exhibit diverse local configurations; ordered atomic polyhedra within the nearest-neighbor range are generally defined as short-range order (SRO) [28,29]. Beyond the nearest-neighbor interaction, medium-range order (MRO) is constructed by networked icosahedral cluster assemblies [28]. Icosahedral units interconnect through vertex-sharing, edge-sharing, face-sharing, and interpenetration, forming four typical icosahedral configurations, among which interpenetrating icosahedra dominate statistically in amorphous matrices [28]. The characteristic atomic clusters formed by interpenetrating icosahedra are illustrated in Figure 1a. Icosahedral bonds link the central atoms of adjacent interpenetrating clusters; longer icosahedral bonds correspond to more fully developed interpenetrating structures, ultimately constructing a continuous network-like icosahedral MRO framework.

For CuZr-based BMGs, Cu-centered full icosahedra (FI) represent the most abundant polyhedral structural motifs and strongly correlate with the mechanical properties of the amorphous matrix. Interpenetrating FI with a Voronoi index <0,0,12,0> permeate the entire structure and form the network-like icosahedral MRO (Figure 1b). Featuring dense atomic packing and high structural stability, FI clusters exhibit strong resistance to shear transformation (ST) events. In practice, due to local structural heterogeneity, ST events concentrate in localized regions under external stress and gradually evolve into SBs. During SB formation, FI structures are disrupted and transformed into fragmented clusters. Such structural softening further facilitates shear localization, since fragmented atomic clusters are prone to reconfigure under continuous loading [15,16,30]. Accordingly, MGs with a high fraction of fragmented clusters exhibit highly unstable shear banding and brittle failure during plastic deformation. Dynamic relaxation behavior strongly dominates the atomic mobility and viscoelastic deformation of MGs. Xing et al. established an intrinsic correlation between relaxation heterogeneity and macroscopic viscoelastic response, revealing the decoupling mechanism of α and β relaxations that governs local atomic rearrangement and early plastic deformation [26]. In MG systems, relaxation heterogeneity refers to the spatial inhomogeneity of atomic relaxation dynamics derived from the disordered amorphous structure, where fast-relaxing soft domains and slow-relaxing hard domains coexist within the matrix. Such heterogeneous relaxation behavior fundamentally determines the macroscopic viscoelasticity of MGs, which manifests as the time-dependent elastic and viscous coupled deformation under external loading. Notably, the dynamic evolution of relaxation heterogeneity strongly interacts with shear banding behavior. The active β-relaxation in soft domains provides preferential sites for the nucleation of embryonic SBs, while the constrained relaxation behavior of hard domains hinders the continuous propagation of SBs. In turn, the initiation and rapid propagation of SBs further aggravate structural relaxation imbalance and strain localization, ultimately dominating the macroscopic viscoelastic deformation and fracture characteristics of MGs.

The intrinsic plasticity and brittleness of MGs are fundamentally governed by their inherent atomic structural characteristics and elastic mechanical properties, which can be quantitatively evaluated by the classic Lewandowski criterion proposed by Lewandowski et al. [31]. This criterion establishes a core correlation between the elastic property ratio (μ/B ratio, shear modulus to bulk modulus) and global Poisson’s ratio (ν), clarifying that a high Poisson’s ratio (corresponding to a low μ/B ratio) is an essential prerequisite for MGs to achieve macroscopic tensile plasticity. Such elastic characteristics effectively resist severe shear localization and catastrophic SB propagation, endowing amorphous alloys with homogeneous plastic deformation capability at the macro scale. To further reveal the atomic-scale structural origin of such intrinsic plastic deformation, Ding et al. proposed the structural signature of “soft spots” (also defined as generalized unsaturated matrix sites, GUMs) in MGs [30]. These structurally loose and mechanically compliant atomic domains act as inherent deformation units, which can preferentially bear and redistribute external strain during loading, thereby alleviating strain concentration and inhibiting rapid shear failure. On this basis, minor alloying has been verified as a feasible and efficient intrinsic toughening strategy to regulate the above structural and mechanical behaviors. As systematically demonstrated by Jiang et al., subtle chemical and structural inhomogeneities can be intentionally introduced via reasonable minor alloying modification [34]. This strategy effectively disrupts the densely packed icosahedral short-range order (ISRO) clusters that dominate the rigid matrix of MGs, artificially constructing abundant strain-adaptive soft spots in the amorphous structure. The newly formed soft spots can uniformly disperse localized strain, promote the multiplication and diffusion of SBs, and ultimately realize the optimization of intrinsic plasticity and toughness of BMGs.

### 2.2. Shear Banding

Shear banding describes the full process from SB initiation to propagation, which dominates the room-temperature plasticity and fracture behavior of BMGs [17]. BMGs are generally considered brittle or quasi-brittle materials due to shear banding, a typical form of plastic instability. The term “instability” refers to the autocatalytic, rapid localization of large shear strain. Shear localization acts as a precursor to shear banding. It denotes the concentration of shear strain into local domains upon loading, accompanied by elevated structural disorder and continuous accumulation of free volume; such localized strain concentration will further develop and eventually form mature SBs. Shear banding can be triggered by intrinsic structural features or external stimuli. On the one hand, SBs initiate from the inherent structural heterogeneity of the amorphous matrix. On the other hand, pre-existing stress concentrators such as sample defects and non-uniform strain/stress/displacement fields also act as preferential sites for SB nucleation.

Shear localization is characterized by increased structural disorder and free volume [35,36,37]. At room temperature, SB initiation via shear localization occurs within a timescale ${\Delta t}_{init}$ ≈ 25 μs, and the SB propagation velocity $V_{SB}$ is nearly zero during this brief softening stage [17]. In the plastic deformation stage, SB propagation takes place, with a total propagation time ${\Delta t}_{prop}$ ≈ 1 ms for bulk samples [17]. SB propagation exhibits strong size dependence: the timescale extends to 20–100 ms when the sample dimension decreases to the micro/nanoscale [38]. Without external constraints, SB propagation is a rapid process without steady-state flow. Traditionally, shear banding is regarded as the precursor to fracture in BMGs, as it induces catastrophic failure along a single dominant SB [6].

Without constraints, SBs initiate, accelerate continuously, and quickly develop into cracks that cause catastrophic fracture. As a result, the tensile plastic strain of MGs is nearly zero, and uniform elongation is unachievable [21,22]. Therefore, obtaining reliable tensile ductility is a prerequisite for the engineering application of MGs. Numerous studies have focused on improving the deformability of MGs via various synthesis routes or post-fabrication treatments [24,25]. These studies primarily optimize the microstructural configuration of MGs by introducing crystalline phases or constructing dual-phase structures, which can effectively modulate the nucleation and propagation of SBs, alleviate shear localization, and ultimately enhance the overall plastic deformability of MGs. Recent in situ observations have revealed that SB propagation exhibits intermittent “stop-go” behavior in hyperquenched MGs, which provides a new structural basis for controllable shear banding [39]. The essence of shear banding is closely associated with the evolution of microscopic structural defects. Chen et al. proposed a unified theoretical model combining quasi-point defects and fractional viscoelasticity, which effectively describes the dynamic evolution of free volume and clustered defects during shear band initiation and propagation [33]. Against this research background, this review concentrates on the mainstream toughening route for MGs, namely regulating shear banding behavior to achieve improved ductility, including suppressing SB initiation and slowing down its accelerated propagation. Relevant strategies will be elaborated systematically in the following sections.

## 3. The Strategies to Enhance the Ductility of MG

This section classifies toughening strategies into two categories based on their physical mechanisms: preventing SB initiation and restraining SB propagation. Both approaches alleviate strain localization and prevent the formation of catastrophic shear bands. Nanosizing and metamaterial design primarily prevent SB initiation, promoting widespread STZ activation and a high deformation participation ratio. Nanosizing refers to a representative strategy that reduces the characteristic dimension of MGs down to the nanoscale, below the critical size for mature SB formation, so as to suppress shear localization and enable homogeneous plastic deformation. Metamaterial strategies involve the rational design of internal micro-architectures of MGs, through which the nucleation and propagation of SBs can be regulated, thereby enhancing tensile ductility without relying on compositional changes. MG matrix composites (MGMCs), nanoglass (NG), notched design, and rejuvenation mainly restrain SB propagation by reducing the SB velocity. Notched design refers to a strategy that introduces prefabricated geometric notches into MG samples to alter the local stress state and stress gradient. This intentional configuration suppresses unstable SB propagation, promotes the activation of multiple SBs, and improves tensile ductility and fracture toughness. It is a typical geometrical toughening approach for MGs.

### 3.1. Prevent Shear-Band Initiation

#### 3.1.1. Nanosized

The size effect indicates that reducing material dimensions to a critical threshold can induce unique mechanical properties. The extrinsic size effect strongly modifies the mechanical behavior of MGs at the nanoscale, enabling the combination of ultrahigh strength and tensile ductility. In 2010, Greer et al. investigated the size effect of Zr_35_Ti_30_Co_6_Be_29_ BMG under tensile loading [40]. The samples with thicknesses ranging from 100 nm to 875 nm are shown in Table 1. The results reveal that BMGs with thicknesses above 100 nm fail catastrophically via a dominant SB without obvious plasticity. In contrast, reducing the size to 100 nm induces a brittle-to-ductile transition while retaining ultrahigh strength. The true stress reaches 2.9 GPa and true strain reaches 25% at a sample diameter of 100 nm. By precisely tailoring sample dimensions, Zr-based MGs can simultaneously deliver ultrahigh strength and excellent deformability [40].

**Figure 2:**
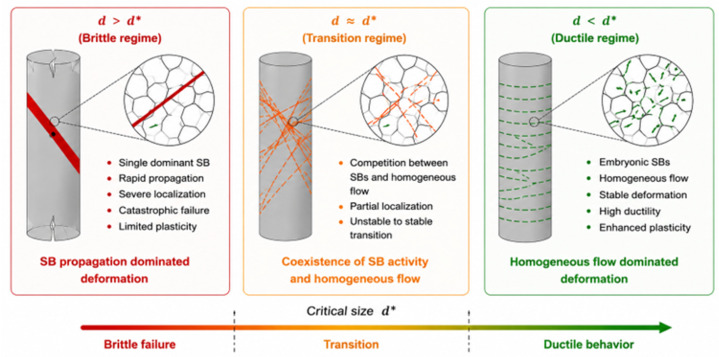
Schematic of size-dependent competition between SB propagation and homogeneous flow in MGs. Above the critical size (d*), deformation is dominated by SBs; below d*, homogeneous flow prevails. Adapted and modified from Ref. [40].

Plastic deformation in MGs involves two competing mechanisms: unstable shear banding and homogeneous flow, whose competitive relationship is highly dimension-dependent [41]. As illustrated in Figure 2, MG nanopillars with diameters larger than the critical size *d*^∗^ fail via rapid SB propagation without notable plasticity. The critical size here refers to the threshold characteristic dimension of MGs, beyond which mature SBs will readily generate. When the diameter is smaller than *d*^∗^, MG nanopillars exhibit nonlinear plasticity after yielding, with deformation dominated by homogeneous flow and embryonic SBs. In addition, sample geometry also plays a vital role in modulating the competition between the above two deformation modes, as systematically demonstrated in our previous study [27]. The variation in geometric characteristics can effectively alter the stress distribution and constraint conditions within the sample, thereby further regulating the dominant deformation mechanism and macroscopic plastic behavior of MGs.

Reducing extrinsic dimensions (i.e., external geometric sizes including sample diameter, thickness, and aspect ratio) below the critical size for mature SB formation suppresses SB initiation, leading to mechanical responses fundamentally different from those of macroscopic BMGs. Notably, extrinsic dimensions are distinct from intrinsic dimensions; the latter refers to the inherent microstructural characteristic scales of MGs (e.g., free volume size and critical shear band thickness) that are determined by material intrinsic atomic configurations, while extrinsic dimensions are artificially tunable macroscopic geometric parameters. The size effect enables homogeneous deformation by transforming shear banding into uniform flow, achieving both high strength and large ductility at room temperature. However, this strategy is limited to nanoscale MG samples and cannot be directly applied to macroscopic components. Recent studies have extended the size effect to gradient nanocrystalline–amorphous composites, achieving ductility retention in submillimeter-scale samples [42].

#### 3.1.2. Mechanical Metamaterials

Mechanical metamaterials are artificial structures with counterintuitive mechanical properties not found in nature. Typical characteristics include low density, controllable stiffness, zero or negative Poisson’s ratio, vanishing shear modulus, and negative compressibility, all governed by structural geometry rather than intrinsic material properties. As a key mechanical parameter, Poisson’s ratio is defined as the negative ratio of the transverse strain to the axial strain under uniaxial loading. For MGs, Poisson’s ratio is closely correlated with atomic packing state, structural flexibility and shear deformation behavior. A higher Poisson’s ratio corresponds to enhanced atomic mobility and improved shear deformability, which facilitates the nucleation of embryonic SBs; in contrast, structural design that tailors Poisson’s ratio can effectively regulate strain localization and restrain the rapid propagation of dominant shear bands, thereby optimizing the tensile ductility and fracture toughness of MG systems. Based on structural design, high-toughness, lightweight MG-based mechanical metamaterials are divided into two categories: nanolattice metamaterials and chiral metamaterials [43].

##### Nanolattice Metamaterials

Nanoscale MGs can exhibit exceptional ductility, but such favorable strain-hardening behavior is limited to the nanoregime. Nanolattice metamaterials are a novel class of mechanical metamaterials whose effective properties depend on both lattice architecture and the wall thickness of hollow members, namely hollow structural units forming the lattice framework. MG nanolattices can scale nanoscale ductility to macroscopic dimensions.

**Figure 3:**
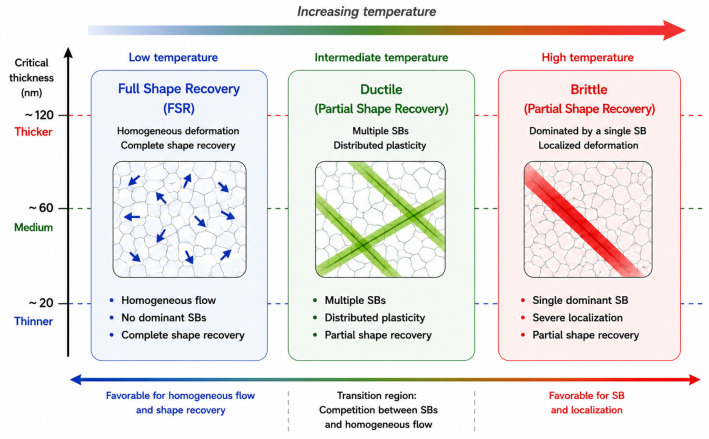
Temperature–thickness deformation map showing brittle, ductile, and full-shape-recovery regimes in MGs, with arrows denoting the increasing direction of temperature (horizontal) and critical thickness (vertical), as well as the transition trend between homogeneous deformation and shear band-dominated deformation at the bottom. Adapted and modified from Ref. [43].

In 2015, Lee et al. fabricated the hollow-tube MG nanolattices via radio-frequency magnetron co-deposition of Cu and Zr, integrating the advantages of nanomaterials and lattice architectures [43]. The overall dimensions reach submillimeter scale, while the tube-wall thickness remains nanoscale. Table 2 summarizes the wall-thickness-dependent deformation mechanisms and fracture behaviors of hollow-tube Cu_60_Zr_40_ MG nanolattices at 298 K. The 120 nm-thick samples show catastrophic layer-by-layer collapse, while the 60 nm-thick samples exhibit smooth plastic flow, a continuous and stable deformation mode enabled by homogeneous SB evolution, or local rather than global brittle fracture. Global brittle fracture refers to the overall sudden failure of the entire sample triggered by the rapid propagation of a single dominant SB across the whole structure. In contrast, local brittle fracture only occurs in partial regions and will not lead to complete structural collapse. For MGs, plastic flow corresponds to the uniform and sustained shear rearrangement of atomic clusters, rather than unstable localized shear deformation. Achieving homogeneous plastic flow is of critical importance because it can effectively suppress strain localization and catastrophic shear fracture, thereby enabling reliable tensile ductility and broadening the structural application potential of MGs. A brittle-to-ductile transition occurs with decreasing wall thickness. Here, the “brittle” means catastrophic fracture of the layers without any initiation of SB. Figure 3 constructs the deformation mechanism map, indicating that the mechanical behavior of hollow-tube MG nanolattices can be tuned by temperature and wall thickness. At 298 K, a transition from partial shape recovery (PSR) to full shape recovery (FSR) occurs between 60 nm and 20 nm wall thickness. PSR and FSR are dominated by plastic deformation and pure elastic deformation, respectively. The excellent ductility mainly stems from shell buckling of hollow tubes, which forms low-stress hinges that prevent SB initiation and accommodate large reversible macroscopic strain. The nanoscale wall thickness imposes a strong size confinement effect, which restricts the nucleation and penetration of mature shear bands and avoids catastrophic shear localization. At 130 K, the deformation mode directly shifts from plastic to elastic without an intermediate transition [43]. Low temperature suppresses atomic rearrangement and shear transformation activities of the MG matrix, further inhibiting SB initiation and thus reducing plastic deformation capacity. Therefore, the plasticity of thinner-walled samples arises from local brittle fracture rather than global homogeneous deformation. Although local failure may occur, hollow-tube MG nanolattices can recover globally if the damage cannot propagate through the entire hollow member [43].

MG nanolattices with nanoscale wall thickness exhibit mechanical properties distinct from bulk BMGs and show excellent toughness. However, localized bending deformation at hollow nodes inevitably induces stress concentration during loading, which renders hollow-tube nanolattices relatively lower strength than bulk counterparts. Geometric discontinuity and structural mutation at hollow nodal positions break the uniform stress field under external loading, resulting in significant local stress concentration. Such concentrated stress preferentially activates atomic rearrangement and shear transformation zones within the nodal region, thereby triggering the preferential initiation and localized propagation of SBs. This node-dominated shear banding behavior is commonly observed in MG nanolattices, which further deteriorates the structural load-bearing capacity and macroscopic strength.

##### Chiral Metamaterials

A chiral structure cannot be superimposed onto its mirror image via rotation or translation. Chiral design is the primary approach to introduce auxeticity (negative Poisson’s ratio) into materials. The term auxeticity is derived from the Greek word *auxetikos*, referring to the characteristic that structures or materials exhibit a negative Poisson’s ratio. Conventionally, chiral nanomaterials are widely associated with molecular chiral ordering and liquid crystal systems, where their chiral topological features originate from the ordered arrangement of molecular units and liquid crystal phase transformation behavior at the microscale. Differently, the chiral MG metamaterials discussed in this work achieve intrinsic structural chirality purely through macroscopic geometric configuration design. Such structural chirality does not rely on molecular-level chiral ordering, liquid crystal characteristics, or chemical asymmetry, but exclusively depends on the tailored topological geometry of the structural unit. This unique structural feature endows MG chiral metamaterials with stable auxetic mechanical responses and distinctive deformation mechanisms different from traditional chiral nanomaterials. Accordingly, chiral structures can be employed to construct mechanical metamaterials with negative Poisson’s ratio. Sha et al. proposed a Cu_50_Zr_50_ MG chiral nanolattice (MGCN) with hexagonal symmetry to achieve negative Poisson’s ratio and enhance functionality [44]. The equation shows initial Poisson’s ratio $\nu_{i}$, where $L=\sqrt{R^{2}-{\left(2\bar{r}\right)}^{2}}$,$\bar{\;r}=r-t/2$, β is the tilt angle of the ligaments, and R is the distance between adjacent node centers. Here, *r* is the node radius, and *t* is the ligament thickness.

Table 3 shows the key mechanical features of Cu_50_Zr_50_ MGCN metamaterials, revealing three deformation regimes. The elastic deformation regime extends to ~9%, demonstrating large elastic deformability [44]. The plastic deformation regime reaches 24.8% tensile strain, with obvious strain hardening after yielding. Fracture occurs at 27.5% strain. The ultimate tensile strength of MGCN is ~0.8 GPa, much lower than the 3.2 GPa of monolithic BMGs [44]. In the elastic regime, Poisson’s ratio ν ≈ −0.65, corresponding to auxetic behavior driven by node rotation under non-central forces. In the plastic regime, Poisson’s ratio increases sharply, reaching zero at 21.5% strain and becoming positive with further deformation. The sign change of Poisson’s ratio depends on rotational units and non-affine deformation kinematics, accompanied by a brittle-to-ductile transition [45]. Accompanied by the change of Poisson’s ratio, the plastic deformation of MGCN metamaterials shifts from brittle to ductile. Atomic deformation snapshots reveal the deformation mechanism: Atomic deformation snapshots refer to instantaneous atomic configuration images captured at different deformation stages via molecular dynamics simulation. These visual data intuitively present the real-time position changes, rearrangement behaviors and stress distribution of atoms within the material during loading. In the elastic regime, non-central forces induce node rotation and ligament bending; in the plastic regime, as non-central forces diminish, ligament deformation shifts from bending-dominated to stretching-dominated. Throughout the deformation process, the nanoscale ligament design effectively suppresses the initiation of SBs.

Throughout deformation, nanoscale ligament design suppresses SB initiation. Chiral structural design produces MGCN metamaterials with large elastic deformation, low density, and excellent ductility. However, these superior properties are achieved at the cost of reduced Young’s modulus and strength.

### 3.2. Restrain SB Propagation

#### 3.2.1. Metallic Glass Matrix Composites (MGMCs)

**Figure 4:**
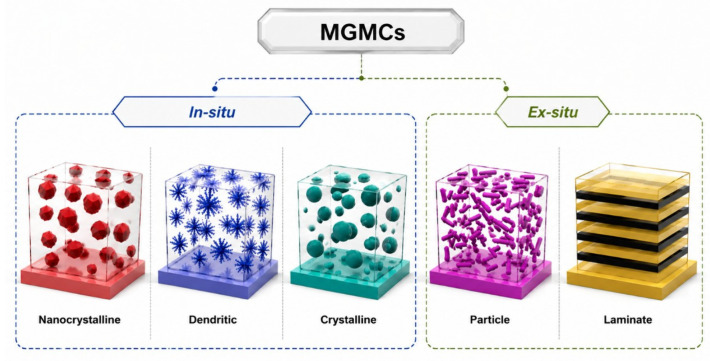
Classification of MGMCs.

The most straightforward approach to overcome the brittleness of monolithic MGs is to introduce a secondary phase, which integrates the advantages of multiple components. Figure 4 classifies MGMCs based on the reinforcement phase within the glass matrix. The continuous MG phase is defined as the matrix regardless of the volume fraction of the secondary phase. MGMCs are divided into in situ and ex situ composites based on reinforcement formation [46,47,48]. Following thermal treatment, metastable MGs undergo local crystallization to form in situ MGMCs. The thermal treatment for inducing local crystallization is generally implemented within the supercooled liquid region of MGs. Temperature exerts a prominent effect on shear banding behavior across different ranges. At room temperature, SBs propagate rapidly in a highly localized manner and easily trigger brittle fracture. Within the supercooled liquid region, elevated temperature increases atomic mobility and free volume content, slowing down SB propagation and facilitating the generation of multiple SBs. Excessively high temperatures beyond this range will trigger full crystallization of the amorphous matrix and completely alter the deformation mode. In situ composites are formed by local crystallization of metastable MGs during thermal treatment, including nanocrystallites, dendrites, and crystalline phases. Ex situ composites employ artificially assembled reinforcements such as particles, polymers, and amorphous alloys. These strategies retard rapid SB propagation and promote the formation of multiple SBs.

Ex situ polymer-reinforced MGMCs were first successfully synthesized by Kim et al. [47]. Incorporating polyisoprene into CuZr MG improved plastic flow by >30% compared with pure CuZr MG without strength loss. The enhanced ductility is primarily attributed to the introduced nanoscale polymer layers. Benefiting from the nanoscale size effect and intrinsic damping capability, these interfacial polymer layers effectively hinder rapid SB propagation, dissipate localized deformation energy, and promote stable plastic flow, thereby substantially improving the tensile ductility of the composite. Polymer layers impede the rapid propagation of single SBs and force new SBs to nucleate at less favorable sites. In contrast, in situ MGMCs attract extensive attention due to simple casting and high glass-forming ability (GFA) of the matrix. Hofmann et al. experimentally verified this strategy: by adjusting alloy composition and semi-solid processing, MGMCs with different dendritic volume fractions were fabricated [48]. The heterogeneous dendritic microstructure prevents catastrophic failure caused by unrestricted SB extension and improves global plasticity. The essence of ductility enhancement is that the second phase retards shear banding and induces multiple SB formation, which acts as a form of work hardening.

Ductile crystalline reinforcements significantly improve the intrinsic brittleness of monolithic BMGs. Unlike the highly localized deformation of monolithic BMGs, second-phase-reinforced MGs suppress accelerated SB propagation. However, introducing a second phase may reduce the GFA of the BMG matrix. Liquid-phase stability and crystallization resistance determine amorphous formation; slight composition variations may cause full crystallization of the glass matrix. Therefore, the second phase and its composition must be precisely controlled. Double-network MG composites with ductile alloy matrices have achieved balanced strength–ductility without severe GFA reduction [49].

#### 3.2.2. Nanoglass (NG)

Grain boundaries strongly affect the mechanical properties of crystalline materials, but BMGs lack grain boundaries due to the absence of long-range order (LRO). LRO refers to the periodic, repetitive, and stable atomic lattice arrangement that persists over a large spatial scale in crystalline materials, which is the essential structural foundation of traditional grain boundaries and dislocation defects. Different from crystalline alloys, BMGs are structurally characterized by short-range atomic ordering but complete absence of LRO, resulting in a disordered glassy matrix without grain boundaries or other typical crystalline defects. Owing to the lack of LRO-dominated crystalline defect systems, conventional grain-based strengthening and toughening mechanisms fail to apply to BMGs. In this context, introducing artificial planar defects provides an effective alternative strategy to regulate atomic structural heterogeneity and tailor the mechanical properties of BMGs. Introducing planar defects into BMGs is an effective approach to tailor mechanical properties. One method is preloading-induced SB formation; another is constructing nanoglasses (NGs) with grain-boundary-like interfaces to regulate the amorphous structure. NGs, a novel amorphous architecture proposed by Jing et al. in 1989, enable effective property tuning of BMGs [50].

**Figure 5:**
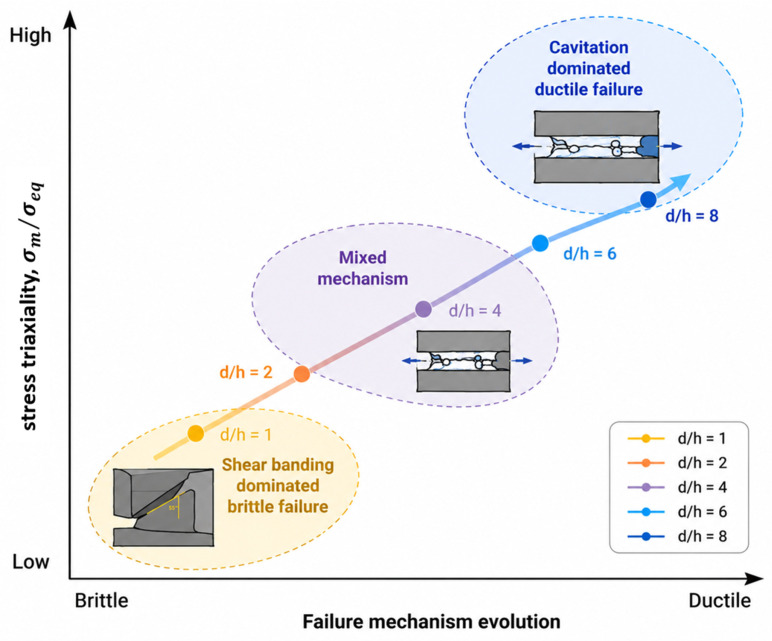
Inverse notch effect governed by a transition from shear banding to void growth in BMG. This is controlled by the ratio of stress triaxiality,$\;\sigma_{m}/\sigma_{eq}$, as a function of the notch dimension, *d*/*h*. Adapted and modified from Ref. [57].

In 2015, Wang et al. fabricated NG via inert gas condensation and monolithic MGs with fully identical chemical composition via melt spinning [51]. NGs consolidated from amorphous nanoparticles exhibit a morphology similar to nanocrystalline alloys. Table 4 summarizes the tensile mechanical properties and deformation behavior of Sc_75_Fe_25_ NG and monolithic MG. The 400 nm Sc_75_Fe_25_ NG shows ~15% plastic strain, while the monolithic MG exhibits negligible plasticity. The uniform tensile plasticity of NGs is attributed to their unique nanostructure, which suppresses single SB propagation and promotes intersecting multiple SBs. The high-resolution TEM inset in Figure 5 clearly shows glassy grains (~10 nm) and glass–glass interfaces (GGIs, ~1 nm). GGIs are soft regions with higher free volume, while MG grains are hard regions. Ritter et al. confirmed that GGIs contain 1~2% excess free volume, with structure and properties similar to preloaded SBs [52]. BMGs with planar defects remain stable at elevated temperatures or under pressure without topological changes [52].

Adibi et al. investigate the grain-size dependence of tensile fracture in Cu_50_Zr_50_ NGs [53]. The discussed grain size refers to the characteristic size of amorphous grains inside the NG. Smaller amorphous grains can constrain SB propagation, promote multiple SB nucleation and alleviate localized deformation, while larger grains tend to induce rapid SB expansion and premature fracture. As the grain size decreases, the deformation mode gradually transitions from a dominant single shear band (*d* ≈ 15–10 nm) to cooperative shear-band-mediated deformation (*d* ≈ 10–5 nm), and finally to homogeneous superplastic flow (*d* ≤ 5 nm). In contrast to monolithic MGs, which typically exhibit brittle tensile fracture, NGs show significantly enhanced deformability with decreasing grain size. These results demonstrate that the mechanical properties of NGs can be effectively tailored by controlling the glassy grain size and the thickness of glass–glass interfaces (GGIs), providing an additional strategy for optimizing the strength–ductility balance of MGs.

Nanoscale structural heterogeneity severely limits SB propagation paths. SBs formed in soft GGIs are blocked by hard grains, retarding accelerated propagation and endowing NGs with excellent tensile plasticity. Importantly, such plasticity enhancement is not merely attributed to the physical blocking effect of refined glassy grains. The heterogeneous hard–soft microstructure enables the sustainable activation and multiplication of numerous minor SBs during deformation, rather than the rapid growth of a single dominant SSB. The continuous proliferation and interaction of multiple SBs consume excessive strain energy and produce a notable strain-hardening effect, which counteracts the intrinsic strain softening of amorphous materials, stabilizes macroscopic uniform plastic flow, and further suppresses catastrophic tensile fracture. However, the high plasticity of NGs is accompanied by severely reduced yield strength, limiting widespread engineering application.

#### 3.2.3. Notched

The notch effect on the strength and plasticity of crystalline materials is governed by dislocation-mediated deformation [54]. Brittle materials typically show notch weakening, while ductile materials show notch strengthening. For example, notched amorphous ceramics exhibit reduced strength compared with un-notched samples, while ductile metals and intermetallics show notch strengthening. Owing to the absence of dislocation activity, BMGs exhibit fundamentally different notch responses from crystalline materials [54,55].

Gao et al. systematically investigated the notch effect on the mechanical performance of Zr-based BMGs through both experimental tests and numerical simulations using symmetric double-edge notched specimens [56,57]. Their parametric study quantitatively revealed the influence of double-edge notch geometric parameters on the tensile strength and ductility of BMGs. Table 5 summarizes the tensile mechanical responses of Zr-based BMG specimens with varying notch geometries, as well as the un-notched sample, revealing the effect of the *d*/*h* ratio (residual cross-sectional diameter/notch height) on strength and ductility. The variation in the *d*/*h* ratio induces a distinct transition in material deformation mechanisms. Specifically, a low *d*/*h* ratio of 1 leads to catastrophic brittle fracture dominated by a single major SB. In contrast, when the *d*/*h* ratio increases to 6, the deformation mode transforms significantly, featuring uniform tensile elongation governed by abundant multiple SBs. The typical dimples and microcracks observed on the corresponding fracture surfaces further verify the formation of multi-SB deformation characteristics. At *d*/*h* = 6, fracture strength reaches 2.9 GPa and tensile plastic elongation reaches 10%, indicating simultaneous enhancement of strength and plasticity. Notch strengthening means that notches increase rather than decrease material strength.

The notch-strengthening effect indicates that the strength of a material is significantly enhanced rather than reduced by the introduction of a notch. The notch effect on the strength of BMGs remains controversial: notch strengthening, notch weakening, and notch insensitivity have all been reported. These controversies are governed by the notch configuration and overall geometry of BMG samples [58]. Notches transform the uniaxial stress state into a triaxial stress state, switching the failure mode. Pan et al. rationalized these controversies using a stress-state model [57].

(1) $$\frac{\sigma_{m}}{\sigma_{eq}}=\frac{1}{3}+\ln\left(1+\frac{d}{2h}\right)$$

where $\sigma_{m}$ is the hydrostatic stress, $\sigma_{eq}$ is the von Mises equivalent stress. This equation is used to describe the stress triaxiality level at the notch center. According to Equation (1), the logarithmic term equals zero for un-notched samples, and shear banding dominates the entire deformation process. For notched samples, $\sigma_{m}$/$\sigma_{eq}$ increases with *d*/*h*. When $\sigma_{m}$/$\sigma_{eq}$ reaches the threshold value $\sigma_{c}$/$\sigma_{s}$, the failure mode switches from shear banding to void nucleation, growth, and coalescence (see Figure 5). Here, $\sigma_{c}$ and $\sigma_{s}$ denote the critical stresses for cavitation and shear localization, respectively. The critical stress triaxiality ratio $\sigma_{c}$/$\sigma_{s}$ is calculated to be approximately 1.4. Based on Equation (1), the stress triaxiality at *d*/*h* = 1 is 0.74, which is below the critical value; thus, the sample is expected to fail via shear localization, as shown in Figure 5. In contrast, the stress triaxiality at *d*/*h* = 6 is 1.72, exceeding the threshold, and deformation proceeds via void nucleation and growth, thereby producing a measurable tensile ductility. The inverse notch effect arises when the stress triaxiality ahead of the notch exceeds the critical threshold.

Qu et al. characterized the notch geometry effect using the stress concentration factor K_t_ [59]: as K_t_ decrease, the notch strength ration (NSR) increases. NSR is defined as the ratio of the ultimate tensile strength of notched to un-notched samples: NSR < 1 indicates notch weakening, NSR > 1 indicates strengthening, and NSR ≈ 1 indicates insensitivity [55]. In contrast to the strength effect, notch-induced ductility enhancement is consistent. The mechanism is that notch-induced stress concentration promotes multiple SB initiation, and the stress gradient impedes rapid SB propagation. BMGs exhibit unique notch strengthening and toughening, distinct from conventional materials.

**Figure 6:**
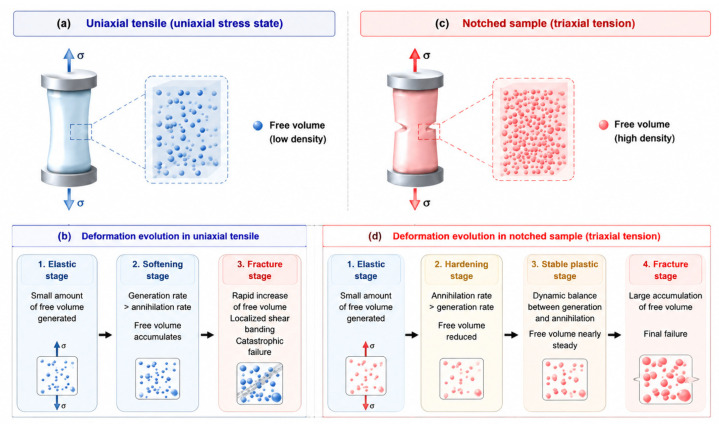
Effect of stress state on free volume evolution and mechanical behavior in BMGs. Adapted and modified from Ref. [58].

From a structural evolution perspective, the runaway failure of BMGs arises from free volume accumulation exceeding annihilation. Wang et al. revealed that notches reverse this trend: free volume annihilation outpaces accumulation [58]. Using the free volume model, they demonstrated that the stress-state change at the notch center reflects a competition between free volume generation and annihilation. As shown in Figure 6a–d, the stress state significantly affects the free volume generation and annihilation rates in a BMG under tension. In Figure 6a,b, the free volume generation rate of a BMG under uniaxial tension is much higher than its annihilation rate. Consequently, the mechanical behavior of the BMG is dominated by the rapid propagation of a single SB, accompanied by strain softening and catastrophic failure. The introduction of a notch modifies the stress state and suppresses strain softening. From Figure 6c,d, a regime can be observed in which the free volume annihilation rate exceeds its generation rate, corresponding to strain hardening and appreciable plasticity. The competing kinetics of free volume accumulation and annihilation indicate that the introduction of a notch leads to hardening rather than softening in BMGs.

#### 3.2.4. Rejuvenation

**Figure 7:**
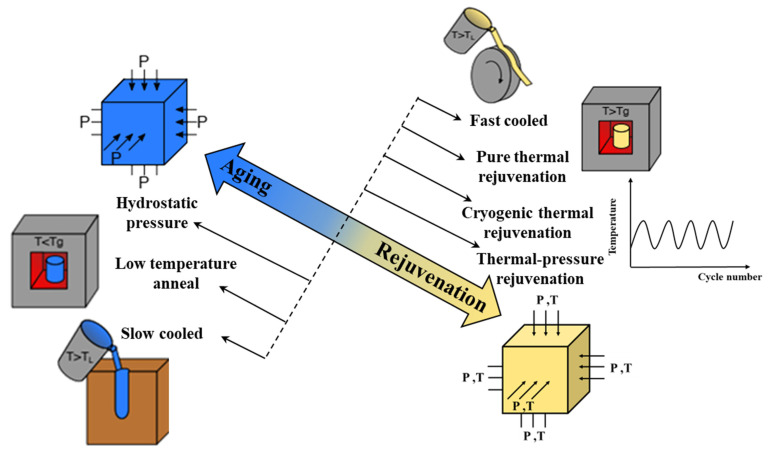
Schematic energy evolution and structural characteristics of glass rejuvenation and aging. The vertical axis denotes the system energy (increasing upward). From left to right, the structural diagrams correspond to the rejuvenated glass, initial amorphous glass, and aged glass, respectively.

Glasses relax to lower-energy states via extremely slow flow, a process called aging [6]. Structural relaxation/aging can be induced by slow cooling, annealing, or pressure application, altering most physical, chemical, and mechanical properties. Rejuvenation refers to driving the glassy system away from the low-energy relaxed state via heating or mechanical stress, partially reversing aging and increasing the energy and volume of the glass. The ability to control aging and rejuvenation is critical for the functional application of BMGs. Figure 7 schematically illustrates rejuvenation and aging, including mechanical processing, pure thermal, and pressure-promoted thermal routes.

##### Mechanical Processing

BMGs can be rejuvenated to a higher energy state via mechanical [60,61,62,63,64] or thermal treatments [65,66,67,68] by introducing structural defects, effectively regulating mechanical properties. Common room-temperature mechanical methods to improve the intrinsic brittleness of monolithic BMGs include rolling [60], wire-drawing [61], shot peening [62], imprinting [63] and high-pressure torsion (HPT) [64]. Rolling achieves high strain via repeated passes, producing a maximum tensile plastic strain of 0.27 ± 0.04% with work hardening [60]. Multi-pass wire drawing achieves a cross-sectional area reduction of ~93% and improves macroscopic ductility [61]. Shot peening provides surface modification [62]; mechanical surface defects alter structure and properties and improve plastic flow. Imprinting creates periodic soft–hard heterogeneous patterns on the sample surface [63], retarding SB propagation and enhancing tensile ductility. HPT reduces the STZ activation energy barrier, enabling the highest plastic strain. N. Adachi et al. tested as-cast and HPT-processed Zr_50_Cu_40_Al_10_ BMGs [64]; HPT improved tensile properties, with tensile plastic elongation reaching 0.34%. Mechanically induced unstable defects increase activated STZs and promote multiple SB formation, defined as structural rejuvenation.

The above-mentioned rolling, wire-drawing, shot peening, imprinting and high-pressure torsion are the adopted cold-working methods. These cold-working methods achieve room-temperature rejuvenation but inevitably cause significant shape change and heterogeneous property distribution, limiting practical application. Ultrasonic vibration treatment has emerged as a low-distortion mechanical rejuvenation method, effectively improving tensile plasticity without shape change [69].

##### Thermal Treatments

In addition to mechanical methods, thermal treatment is an effective and controllable approach to tune MG properties. Pure thermal rejuvenation involves annealing above the glass transition temperature ($T_{g}$) followed by rapid quenching back to the glassy state, restoring plasticity in annealed embrittled samples. The glass transition temperature ($T_{g}$) is a critical thermodynamic threshold that distinguishes the glassy state and supercooled liquid state of amorphous materials. Heating above provides sufficient atomic mobility to rearrange the disordered glassy structure, eliminate residual structural heterogeneity, and induce structural rejuvenation, while subsequent rapid quenching locks the high-energy disordered configuration. Such thermal regulation effectively modulates shear banding behavior and recovers the deteriorated plasticity of embrittled BMGs. This process enables the recovery of plasticity in annealed embrittled samples. However, pure thermal rejuvenation requires a higher cooling rate ($R_{a}$) than the initial melt-quenching rate ($R_{q}$) for glass formation; moreover, it may degrade the intrinsic strength and elastic stiffness [65]. To address these drawbacks, pressure-promoted thermal rejuvenation has been proposed, in which external pressure is imposed during the quenching step of glass formation. When the cooling rate is equal to the initial quenching rate, Miyazaki et al. proposed that pressure-promoted thermal rejuvenation can only be achieved under compressive pressure at relatively high annealing temperatures ($T_{a}>1.1\sim 1.2T_{g}$), whereas tensile pressure accelerates physical aging [66]. However, Li et al. confirmed that pressure-promoted thermal rejuvenation can be realized under compressive pressure when the cooling rate after isothermal annealing is higher than the initial quenching rate [67].

Wakeda et al. revealed that high energy retention arises from the coupling of high temperature and high pressure [65]. In MG systems, high energy retention refers to the phenomenon whereby a large number of high-energy disordered atomic configurations and residual free volume generated under extreme thermo-mechanical coupling conditions are stably locked inside the glassy matrix. This retained high-energy structural state corresponds to structural rejuvenation, which effectively optimizes SB propagation behavior and improves the macroscopic ductility of BMGs. Low pressure induces aging, while high pressure coupled with high temperature induces rejuvenation. Pressure-promoted rejuvenation increases density and SRO, revising the conventional energy–structure relationship. Pressure-promoted thermally rejuvenated MGs are activated into a higher-energy glassy state, thereby tailoring the mechanical properties of MGs. Returning the glass structure to a higher-energy state retains more fragmented clusters and leaves more extensive liquid-like regions. These pervasive liquid-like regions facilitate distributed ST events, which impede the rapid propagation of SBs.

In fact, both pure thermal rejuvenation and pressure-promoted thermal rejuvenation require rigorous and stringent conditions. In contrast, low-temperature thermal cycling rejuvenation effectively circumvents these inherent drawbacks [68]. Conventionally, prolonged structural relaxation of MGs at cryogenic temperatures typically leads to a pronounced deterioration in tensile ductility. Nonetheless, Wang et al. demonstrated that subjecting MGs to repeated thermal cycling between room temperature and liquid nitrogen temperature (77 K) can significantly alleviate room-temperature brittleness [68]. This low-temperature thermal cycling rejuvenation technique eliminates the need for high temperatures and high pressures, making it considerably more feasible for practical engineering applications. However, the rejuvenation efficiency of this method strongly depends on a variety of key parameters, including the number of thermal cycles, cyclic loading rate, and chemical composition of the MGs.

Thermally induced structural rejuvenation stands out for its capability to realize localized structural modification, which can be achieved by means such as transient laser heating. Thermal rejuvenation refers to the technique that adjusts the atomic configuration and energy state of BMGs through heating and subsequent cooling processes, so as to optimize their mechanical performance. This approach can be applied to arbitrary sample geometries and is intrinsically isotropic, which is difficult to achieve via mechanical processing. Moreover, pressure-promoted thermally rejuvenated MGs exhibit better feasibility than pure thermally rejuvenated MGs. However, it remains unclear whether pressure-promoted rejuvenation can be realized in bulk samples while retaining their glassy structure. Recently, Greer et al. demonstrated that rejuvenation of BMGs under triaxial compression can tune their mechanical behavior to achieve exceptionally efficient strain hardening [70]. This mechanism yields strain hardening based on a reduction in internal energy rather than an increase. The rejuvenated BMGs maintain a stable glassy structure and suppress shear band propagation, without any size or mechanical constraint limitations.

Beyond the aforementioned macroscopic structural modification strategies, recent research has demonstrated advanced intrinsic structural design approaches to suppress shear banding without introducing macroscopic secondary phases. High-entropy metallic glasses (HEMGs) possess severe atomic-level topological distortion and inherent chemical heterogeneity, which intrinsically disrupt continuous shear pathways and facilitate uniform plastic flow during deformation [71]. In parallel, thin film metallic glasses (TFMGs) are subjected to pronounced geometric confinement imposed by substrates and interfacial interactions. Such constraint effects substantially alter SB dynamics and effectively block the propagation of SBs, leading to distinct deformation characteristics compared with freestanding MG samples [72]. These two emerging material systems further enrich the methodologies for regulating shear banding and improving the tensile ductility of MGs.

## 4. Conclusions and Future Directions

This review systematically summarizes the representative progress of toughening and ductility enhancement strategies for BMGs. Based on comprehensive collation and in-depth analysis of existing studies, this paper generalizes six mainstream structural regulation approaches for optimizing the tensile deformability of BMGs, including nanosizing design, mechanical metamaterial construction, MGMCs, NG fabrication, notch structural design, and structural rejuvenation. By analyzing the intrinsic correlation between each strategy and SB evolution behavior, we conclude a unified microstructural mechanism for BMG toughening: all feasible toughening methods essentially regulate the amorphous atomic structure and external geometric conditions, thereby inhibiting the rapid initiation and unstable propagation of single SBs and activating uniform multiple SB deformation.

Specifically, size reduction and geometric architecture design suppress premature SB failure; heterogeneous structures including secondary phases and NG interfaces disperse localized deformation; and notch-induced stress gradient and rejuvenation-induced high-energy structural states further promote stable plastic flow. From a fundamental perspective, restraining shear banding instability is the decisive factor for achieving excellent strength–ductility synergy of BMGs. Despite abundant advances in toughening strategy development, the ultrafast dynamic evolution and atomic-scale structural origin of shear banding remain elusive. Therefore, clarifying the structural and dynamic evolution mechanism of SBs is still the key research direction for breaking through the brittleness limitation of amorphous alloys in future studies.

Future directions should focus on: (1) scalable multiscale heterogeneous design; (2) high-entropy MG development for intrinsic ductility; (3) in situ dynamic characterization; (4) machine-learning-aided alloy design; (5) stable room-temperature rejuvenation; (6) multifunctional strong–ductile MGs for aerospace and biomedical applications.

## Figures and Tables

**Table 1:** Tensile deformation behavior of Zr_35_Ti_30_Co_6_Be_29_ BMG samples with different characteristic sizes (Data summarized from Greer et al. [40]. Values are approximate and extracted from the reported stress–strain curves).

Sample Size (nm)	Ultimate True Stress (GPa)	Plastic Strain	Dominant Deformation Mode	Mechanical Response	Sample Size (nm)
875	~1.8–2.0	Negligible	Single mature SB	Catastrophic brittle failure	875
568	~2.2	Very limited	SB dominated	Brittle behavior	568
330	~2.2	Limited	SB dominated	Brittle behavior	330
100	~2.9	~25%	Homogeneous flow + embryonic SBs	Ductile behavior with ultrahigh strength	100

**Table 2:** Effect of wall thickness on deformation and fracture behavior of Cu_60_Zr_40_ MG nanolattices at 298 K (adapted from Ref. [43]).

Feature	120 nm Wall Thickness	60 nm Wall Thickness
Stress level	Higher strength	Lower strength
Deformation behavior	Layer-by-layer collapse	Smooth and continuous plastic flow
SB evolution	Single dominant SB	Distributed and homogeneous SB activity
Failure mode	Global brittle fracture	Local brittle fracture
Strain localization	Severe	Significantly suppressed
Plasticity	Limited	Enhanced
Mechanical transition	Brittle regime	Ductile regime

**Table 3:** Key mechanical features of the Cu_50_Zr_50_ MGCN metamaterials (adapted from Ref. [44]).

Stage	Strain Range	Stress (GPa)	Deformation Feature
I: Elastic regime	0 ≤ ε < 9%	0 → ~0.4	Linear elastic deformation
II: Plastic hardening	9% ≤ ε < 24.8%	~0.4 → ~0.8	Non-linear stress increase with strain hardening
III: Fracture	ε = 27.5%	~0.8 → 0	Catastrophic stress drop, final fracture
Peak strength	ε = 24.8%	0.8 GPa	Ultimate engineering stress before failure

**Table 4:** Tensile mechanical properties and deformation behavior of Sc_75_Fe_25_ NG and monolithic MG (adapted from Ref. [51]).

Material Type	Peak Strength (GPa)	Tensile Plastic Strain	Deformation Mechanism
Monolithic MG	~1.8	<1% (negligible)	Single dominant SB, catastrophic brittle fracture
NG	~1.35	~15%	Multiple intersecting SBs, homogeneous-like plastic deformation

**Table 5:** Tensile mechanical properties and deformation mechanisms of Zr-based BMG specimens with different notch geometries, including the un-notched counterpart. (adapted from Ref. [56]).

Specimen Condition	*d/h* Ratio	Fracture Strength (GPa)	Tensile Plastic Elongation	Dominant Deformation Mechanism	Key Feature
Un-notched (A)	-	~1.6	<1%	Single SB propagation	Brittle fracture, no plasticity
Notched (B)	1	~1.65	<1%	Single major SB	Catastrophic brittle failure
Notched (C)	1.5	~2.0	~2%	Dominant single SB	Limited plasticity
Notched (D)	3	~2.25	~5%	Multiple SBs initiation	Moderate plasticity improvement
Notched (E)	4	~2.25	~8%	Intersecting multiple SBs	Significant plasticity enhancement
Notched (F)	6	~2.9	~10%	Abundant multiple SBs	Simultaneous high strength & ductility

Notes: Different letters denote specimens with different notch ratios.

## Data Availability

No new data were created or analyzed in this study. Data sharing is not applicable to this article.
